# Development and validation of the Tobacco Use Individual-level Simulation and Tracking (TwIST) Model

**DOI:** 10.1371/journal.pone.0342083

**Published:** 2026-02-12

**Authors:** Sarah D. Mills, Nicholas Tapp Hughes, Yu Zhang, Kurt M. Ribisl, Christopher A. Wiesen, Jiaqian Fan, Kristen Hassmiller Lich

**Affiliations:** 1 Department of Health Behavior, Gillings School of Global Public Health, University of North Carolina at Chapel Hill, Chapel Hill, North Carolina, United States of America; 2 Lineberger Comprehensive Cancer Center, University of North Carolina at Chapel Hill, Chapel Hill, North Carolina, United States of America; 3 Department of Computer Science, University of North Carolina at Chapel Hill, Chapel Hill, North Carolina, United States of America; 4 Department of Biostatistics, Gillings School of Global Public Health, University of North Carolina at Chapel Hill, Chapel Hill, North Carolina, United States of America; 5 Odum Institute, University of North Carolina at Chapel Hill, Chapel Hill, North Carolina, United States of America; 6 Department of Health Policy and Management, Gillings School of Global Public Health, University of North Carolina at Chapel Hill, Chapel Hill, North Carolina, United States of America; Central Food Technological Research Institute CSIR, INDIA

## Abstract

Simulation models of tobacco use behavior are useful analytic tools for projecting rates of tobacco use over time and identifying priority areas for intervention. This paper presents the Tobacco Use Individual-level Simulation and Tracking (TwIST) Model, an individual-based simulation model of tobacco use in the adult US population. We describe the model structure, data sources and parameters, and, in addition to future projections, compare modeled estimates of smoking prevalence to those from established surveys. The simulated population and model parameter estimates are informed by the Population Assessment of Tobacco and Health Study and other nationally representative datasets. To simulate tobacco use over time, we estimated 2^nd^ order Markov models using multinomial logistic regression. To validate the model, we compared model estimates of tobacco use to data from three national surveys. The model estimates adult cigarette smoking rates will decline from a prevalence of 12.4% (95% uncertainty interval (95% UI): 12.2–12.8%) in 2020 to 9.6% (95% UI: 9.3–9.9%), 9.1% (95% UI: 8.9–9.4%), and 8.7% (95% UI: 8.5–9.0%) in 2030, 2040, and 2050, respectively. From 2020 through 2050, adults living in poverty are estimated to have a cigarette smoking rate 2.1–2.3 times higher than individuals living above the poverty line. The prevalence of menthol cigarette use will decline at a slower rate than the prevalence of non-menthol cigarette use (21% vs. 38% decline). Model projections of cigarette smoking prevalence typically fall within the 95% confidence intervals of prevalence estimates across three national surveys. Overall, the TwIST Model projects cigarette smoking prevalence rates that are similar to real-world estimates. If tobacco use continues based on current patterns, income-based disparities in smoking will persist and a growing proportion of individuals who smoke will use menthol cigarettes, which are known to be harder to quit.

## Introduction

Cigarette smoking rates in the United States (US) have declined markedly for the past several decades [1]. Reductions in smoking are in part due to the implementation of evidence-based tobacco control interventions such as increased excise taxes that raise the price of cigarettes, counter-marketing media campaigns, smoke-free air laws that limit where individuals can smoke, and smoking cessation support such as state quitlines [2]. As smoking prevalence rates reach historic lows, the interventions needed to produce further declines in smoking are likely to change. In addition, although smoking rates in the overall US population have declined, demographic disparities in smoking by race/ethnicity, socioeconomic status, and other characteristics have stubbornly persisted. This suggests the need for novel tobacco control interventions to reduce demographic disparities in smoking [1,3].

In 2009 the Family Smoking Prevention and Tobacco Control Act was signed into law, which gave the US Food and Drug Administration (FDA) regulatory authority over tobacco products [4]. To guide regulatory decision making, the FDA has called for the use of simulation modeling to estimate the public health impacts of potential tobacco control policies [5]. Simulation models can be used to compare tobacco use and health outcomes under status quo and counterfactual policy settings to estimate policy impacts on public health [6]. Several simulation models of tobacco use behavior have been developed for the US population to estimate the impacts of tobacco control policies [6,7].

Simulation models of tobacco use often progress age-sex cohorts of the US population through annual time steps and track tobacco use in aggregate over time [6]. These cohort-based models assess policy impact by simulating the mean responses of homogenous cohorts to policy interventions [8]. Although used widely, these models are not optimal for examining the impact of policies on demographic disparities in tobacco use. Research finds that tobacco use behavior and policy impacts vary not just by age and sex but also by race/ethnicity, income, and several other demographic characteristics [1,9,10]. In contrast to cohort-based models, individual-based simulation models (also called microsimulation models) simulate behavior among heterogenous individuals and generate modeled tobacco use behavior and policy impacts for each individual in the model as opposed to strictly in the total population or among population sub-groups [8]. This allows for the inclusion of variation in tobacco use behavior due to a potentially wider spectrum of demographic characteristics and for the inclusion of stochastic variation in tobacco use trajectories at the individual level [8]. Individual-based models more easily capture variation in participants’ demographic characteristics and how such characteristics affect tobacco use behavior and policy impacts over time [8].

Simulation models of tobacco use behavior also typically adopt the Markov property when simulating tobacco use over time. The Markov property assumes an individual’s future tobacco use behavior is dependent only on their current tobacco use behavior and is independent of their past [6]. This assumption is made to simplify analytic models and because longitudinal data on tobacco use behavior across longer periods are often not available; however, it is inconsistent with the theoretical and empirical understanding of tobacco use behavior [11,12]. Research shows that future tobacco use is dependent not only on current, but also on past tobacco use behavior [11]. Using data across approximately four years of the nationally representative Population Assessment of Tobacco and Health (PATH) Study, Mills et al. [13] found that higher-order Markov models that consider participants’ prior tobacco use when predicting future tobacco use were a better fit to the data than a first-order Markov model that assumes future tobacco use behavior is only dependent on current tobacco use. In that study, transition probabilities between tobacco use states ranged widely dependent on an individual’s tobacco use history, reflecting the important role tobacco use history played in determining tobacco use behavior over time. For example, the estimated percentage of menthol cigarette smokers, non-menthol cigarette smokers, and e-cigarette/dual users who transitioned to former smokers (i.e., quit) one year later ranged widely from 4–58%, 5–60%, and 5–64%, respectively, dependent on tobacco use history [13]. This research suggests simulation models should take tobacco use history into account as much as possible to more accurately reflect real-world tobacco use behavior.

This paper describes the Tobacco Use Individual-level Simulation and Tracking (TwIST) Model, an individual-based simulation model of tobacco use behavior in the US adult population. The TwIST Model improves upon other simulation models of tobacco use in several ways. The Model uses an individual-based modeling approach that can assess differences in smoking prevalence among various demographic groups over time and therefore better capture how demographic subgroups may differentially respond to policies and programs. In addition, the simulation model projects tobacco use behavior using a second-order Markov model where future tobacco use depends on both current and prior-year tobacco use. The model also distinguishes between users of menthol and non-menthol cigarettes. Research finds that individuals are more likely to initiate with menthol cigarettes as compared to non-menthol cigarettes, and menthol cigarettes are more difficult to quit [14,15]. Therefore, modeling tobacco use behavior separately for individuals who smoke menthol and non-menthol cigarettes may more accurately reflect real-world tobacco use behavior and cessation over time (here, modeled through 2050). For model transparency we provide a detailed description of the model structure, data sources, and model parameters. To validate the simulation model, we compare simulated estimates of smoking prevalence to data from established nationally representative surveys. Estimating tobacco use patterns over time, the TwIST model can highlight where tobacco control efforts may need to focus to most effectively reduce tobacco use and related morbidity and mortality in the US and test the impact of potential policy interventions.

## Materials and methods

The TwIST Model is an individual-based simulation model that projects tobacco use in the US adult population. The simulated population and parameter estimates in the model are informed by data from the PATH Study [16] and other nationally representative datasets. We initiated the model in 2016 with a population of US adults aged 18–64 years, using PATH data from 2016 and appropriate weights to project to the full US adult population. In each year after, we incorporate a new cohort of 18-year-olds in the model using PATH data (and associated weights) when available to maintain a full 18–64 year old US adult population over time. PATH data is used to estimate entering cohort size as long as younger cohorts are sampled, with latest cohort size and tobacco use estimates at age 18 projected forward when data availability ends.

In each year of the simulation model, each individual’s tobacco use state is updated or the individual dies (approximately 0.5% of individuals die each year). There are five mutually exclusive tobacco use states to which an individual can be assigned: 1) never smoker, 2) former smoker, 3) menthol cigarette smoker, 4) non-menthol cigarette smoker, or 5) e-cigarette/dual user. See Table 1 for definitions of the tobacco use states. Individuals who only use e-cigarettes and dual e-cigarette and cigarette users were combined into a single category because analysis of PATH data indicated more than half (54% − 62%) of e-cigarette users also used cigarettes.

**Table 1:** Definitions of Tobacco Use States.

Tobacco Use State	Definition
Menthol cigarette smoker	Individual reports having smoked at least 100 cigarettes in their lifetime AND currently smokes some days or everyday AND regular brand/last brand of cigarettes smoked flavored to taste like menthol or mint.
Non-menthol cigarette smoker	Individual reports having smoked at least 100 cigarettes in their lifetime AND currently smokes some days or everyday AND regular brand/last brand of cigarettes smoked is not flavored to taste like menthol or mint.
E-cigarette/dual user	Individual reports ever using an e-cigarette^a^ AND using fairly regularly AND currently using some days or everyday AND is a cigarette smoker (menthol or non-menthol) OR individual reports ever using an e-cigarette^a^ AND using fairly regularly AND currently using some days or everyday.
Former smoker	Individual reports having smoked at least 100 cigarettes in their lifetime AND does not currently smoke some days or everyday.
Never smoker	Individuals reported not smoking at least 100 cigarettes in their lifetime AND not using e-cigarettes fairly regularly AND not currently using e-cigarettes some days or everyday.

^a^In PATH Study Waves 1 and 2, individuals are asked about e-cigarette use. In Waves 3 and 4, individuals report on use of electronic nicotine products and subsequently report on type of electronic nicotine product used. If an individual reported using tank system ‘box style’ e-cigarettes, tank system ‘vapor pen’ e-cigarettes, or disposable ‘cig-a-like’ e-cigarettes, we considered the individual an e-cigarette user.

### Data sources

#### The population and tobacco use behavior.

We used data from the PATH Study to estimate transition probabilities between tobacco use states and to generate a synthetic US adult population for the model. Sponsored by the National Institutes of Health and FDA, PATH is a nationally representative longitudinal cohort study of 13,651 youth (12–17 years) and 32,320 adults (18 + years) that collects detailed self-report data on tobacco use behavior. [16] PATH uses a four-stage, stratified probability sample design to obtain a nationally representative cohort of individuals living in the US [16]. See Hyland et al. [16] for a detailed description of the PATH Study. We used PATH data from Waves 1, 2, 3, and 4, which ended in 2014, 2015, 2016, and 2017, respectively. Participants completed questionnaires during each Wave, which are approximately one year apart [17]. Participant retention across Waves was high with retention rates of 74% to 83% across Waves 1–4 [17].

For this study, we restricted the PATH data to adults who were in Waves 1–4 to obtain a nationally representative sample at the time of Wave 4 (unweighted *N* = 26,072; weighted *N* = 241,543,989). This sample was further restricted to adults 18–64 years old in Wave 1 (unweighted sample size: *N* = 19,357; weighted: *N* = 189,584,407). PATH Study data restrictions regarding small sample sizes prevented us from including individuals of older ages in the simulation model. Each individual in the population is distinguished according to their age, sex, race/ethnicity, age at smoking initiation, poverty status, current tobacco use state, and tobacco use state in the prior year. To minimize levels of missingness on these variables, we used imputed variables created by the PATH Study when developing the initial population. In addition, single value maximum likelihood imputation was used to impute missing poverty status data for 3,155 individuals and missing age data for 14 individuals.

For model calibration we used data from the National Health Interview Survey (NHIS), a nationally representative, cross-sectional survey conducted by the Center for Disease Control and Prevention’s (CDC) National Center for Health Statistics [18]. Using a stratified, multistage probability sampling design of US households, NHIS collects data on a broad range of health topics including tobacco use among the civilian, non-institutionalized US adult population. For model validation, we used data from the Behavioral Risk Factor Surveillance System Survey (BRFSS) and the National Survey on Drug Use and Health (NSDUH) [19,20]. The BRFSS is a cross-sectional, state-and nationally-representative telephone survey coordinated by the CDC that monitors the health behaviors and conditions of US adults [20]. Core BRFSS survey modules include questions about tobacco use. BRFSS uses a complex, probability-based sample design with random-digit dialing of both landline and cell phone numbers [20]. Sponsored by the Substance Abuse and Mental Health Services Administration, NSDUH is a cross-sectional, nationally representative survey that assesses health behavior and outcomes including tobacco use of the non-institutionalized, civilian US population 12 years and older [19]. NSDUH uses an independent, multistage area probability sample and collects data in-person using computer-assisted interviews [19]. For NHIS, BRFSS, and NSDUH, survey weights were applied so that the results are representative of the US adult population. Details about the particular survey years and variables used for model calibration and validation are provided below.

We used the PATH ‘Wave 4: Adult Wave 1 Cohort All-Waves Weights’ to create the initial population and to estimate tobacco use transition probabilities [17]. PATH is a longitudinal cohort study and the PATH study weights are optimal for longitudinal analyses [17]. Comparison of smoking prevalence estimates in the PATH Study to prevalence estimates from the cross-sectional NHIS suggested that PATH-based smoking prevalence estimates for the initial population were over-estimated. Unlike the PATH Study, the NHIS is designed to obtain cross-sectional estimates of smoking prevalence and is considered a gold standard for national data on health behaviors [21,22]. Using PATH data an estimated 18.41% of 18–64 year olds were current cigarette smokers in 2016. According to the NHIS an estimated 15.13% of 18–64 year olds were current cigarette smokers in 2016. To match NHIS smoking prevalence estimates in 2016, we calibrated the smoking prevalence in the initial population of the simulation model by adjusting PATH sample weights. An adjustment factor was used to increase sample weights for non-smokers and to decrease sample weights for smokers while maintaining the original total sum of the weights. PATH Study and other data used for the simulation model were accessed from August 1, 2021 to November 1, 2023. The study team did not have access to information that could identify individual participants.

#### Mortality.

To estimate risk of mortality, we used National Vital Statistics Reports (NVSR) data on mortality risk by age and sex overall and data on mortality risk by smoker status from Thun et al. [23,24] Among individuals 55 years and older, we adjusted death rates, informed by estimates of mortality risk from Thun et al. [23], so that mortality risk each year was lower for never smokers and higher for current and former smokers while maintaining the average death rate by age and sex in the NVSR Life Tables. Additional details are provided below.

#### Model calibration and validation.

To calibrate the model we used NHIS data on the prevalence of cigarette smoking from 2016 to 2020 [18]. For model validation we used data on tobacco use prevalence rates from the BRFSS in 2016, 2017, and 2020 and from the NHIS in 2016–2021 [18,20]. Estimates from the simulation model were not compared to BRFSS data in 2018, 2019, and 2020 because all US states did not include questions about e-cigarette use during that period. We also used data on the prevalence of menthol cigarette use from the NSDUH from 2016 to 2020 [19].

#### Simulating tobacco use behavior.

**Fig 1:**
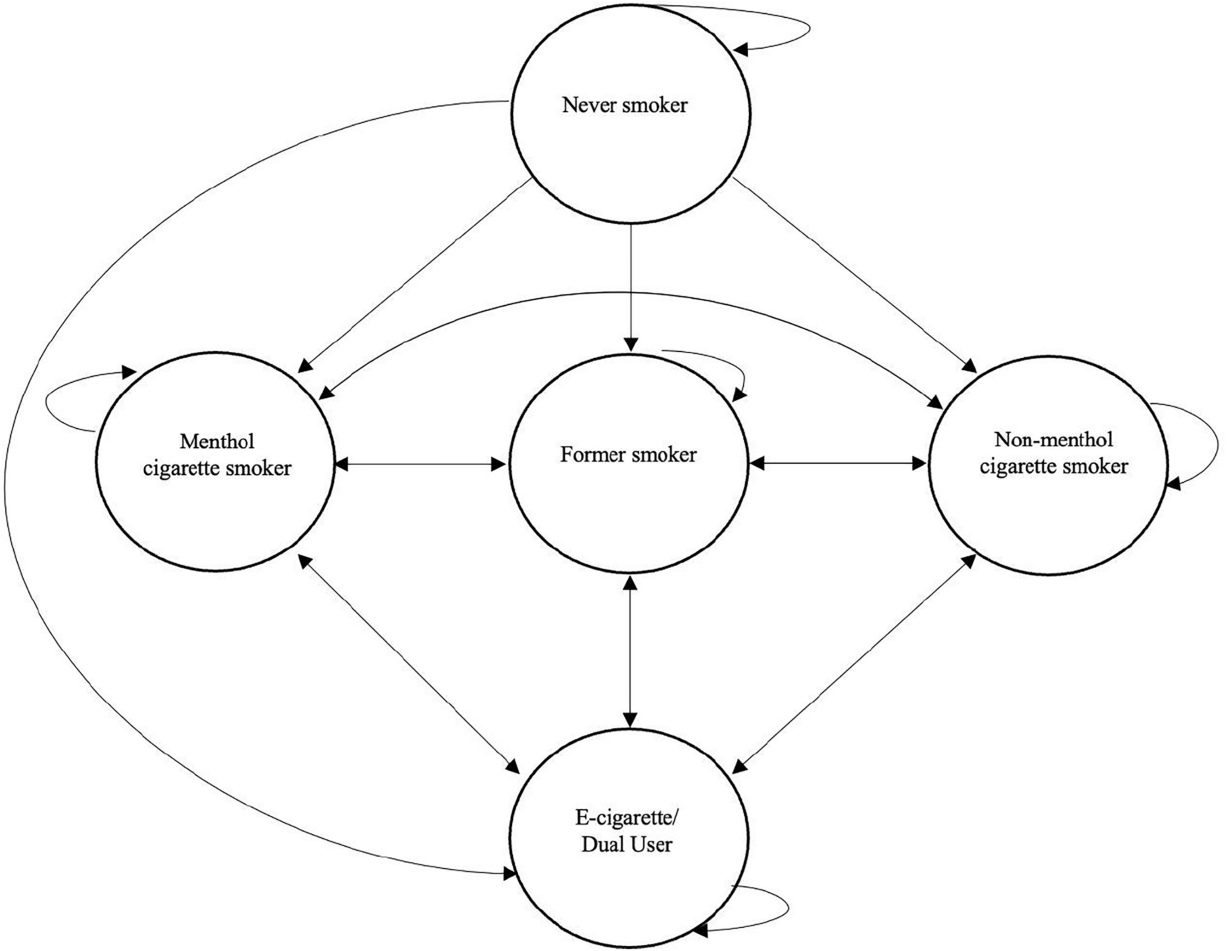
Potential transitions between tobacco use states.

In each year of the simulation model an individual can remain in the same tobacco use state as the prior year (e.g., never smoker in 2023 stays a never smoker in 2024) or transition to a different tobacco use state (e.g., never smoker in 2023 transitions to a menthol cigarette user in 2024). See Fig 1 for all potential transitions between tobacco use states. To simulate tobacco use over time we estimated 2^nd^ order Markov models using two multinomial logistic regression models. One regression model was used to estimate transition probabilities among never-smokers and another was used to estimate transition probabilities among ever-smokers (i.e., former smokers, menthol cigarette smokers, non-menthol cigarette smokers, and e-cigarette/dual users). Two models were estimated because ever-smokers can never transition to never-smokers in a subsequent year and distinct parameter values were therefore needed to estimate transition probabilities for these two groups. This process has been previously described in Mills et al [13].

The regression models estimate the probabilities of individuals in a given tobacco use state transitioning to a subsequent tobacco use state. Simulated transition probabilities between tobacco use states are specific to a participant’s age, sex (male, female), race/ethnicity (non-Hispanic Black, all other race/ethnicities), poverty status (below poverty level, at or above poverty level), smoking initiation age (not smoking, less than 18 years, 18 years or greater), and current and prior-year tobacco use states. Non-Hispanic Black participants self-identified as Black or African American and did not identify as being of Hispanic, Latino, or Spanish origin. Individuals’ demographic characteristics were determined based on their responses to survey questions in Wave 1. The equation for the multinomial regression 2^nd^ order Markov model that estimates tobacco use transition probabilities for ever-smokers is the following:


$$\begin{array}{cc} \;\text{ln}\left(\frac{p_{j,i}}{p_{j,\text{4}}}\right)= & \;\alpha_{i}+\sum_{k=\text{1}}^{\text{4}}\;\beta_{k,i}\times I\;(\text{Tobacco}\;\text{use}\;\text{state}\;\text{at}\;\text{year}\;j-\text{2})\\ & +\sum_{k=\text{5}}^{\text{7}}\;\beta_{k,i}\times I\;(\text{Tobacco}\;\text{use}\;\text{state}\;\text{at}\;\text{year}\;j-\text{1})\\ & \;+{\;\beta }_{\text{8},i}\times I\;(\text{Age}\;\text{when}\;\text{initiated}\;\text{smoking}\;<\text{1}\text{8})\\ & \;+{\;\beta }_{\text{9},i}\times I\;(\text{Age}\;\text{when}\;\text{initiated}\;\text{smoking}\;\geq \text{1}\text{8})\\ & +\;\beta_{\text{1}\text{0},i}\times I\;(\text{Race}\;=\text{White}/\text{Asian}/\text{Hispanic}/\text{Other})\\ & +{\;\beta }_{\text{1}\text{1},i}\times \text{Age}\;\text{in}\;\text{year}\;j\;-\;\text{1}\;\\ & +{\;\beta }_{\text{1}\text{2},i}\times I\;(\text{Sex}\;=\;\text{male})\\ & +{\;\beta }_{\text{1}\text{3},i}\times I\;(\text{Annual}\;\text{household}\;\text{income}\;\text{below}\;\text{poverty}\;\text{level}) \end{array}$$ (A1)

where indicator function I(·)=1 when (·) is true; otherwise I(·= 0. In addition,  *p*_*j,i*_ is the probability of transitioning to tobacco use state *i* in year *j*, and $\beta_{jk,i}\;$ are the regression parameters that correspond to covariate *k* and smoking state *i*. In the model, regression parameters with indices 1 ≤ k ≤ 7 correspond to tobacco use state covariates, whereas those with indices 8 ≤ k ≤ 13 correspond to demographic covariates. Values *i* (tobacco use state) = 1, 2, 3, 4, and 5 refer to never smokers, former smokers, menthol cigarette users, non-menthol cigarette users, and e-cigarette/dual users, respectively. The covariates and their associated parameters in the ever-smoker model are the following:

1. never smoker in year *j* – 2 ($\beta_{\text{1},i}$)
2. former smoker in year *j* – 2 ($\beta_{\text{2},i}$)
3. menthol cigarette user in year *j* – 2 ($\beta_{\text{3},i}$)
4. non-menthol cigarette user in year *j* – 2 ($\beta_{\text{4},i}$)
5. former smoker in year *j – 1* ($\beta_{\text{5},i}$)
6. menthol cigarette user in year *j – 1* ($\beta_{\text{6},i}$)
7. non-menthol cigarette user in year *j – 1* ($\beta_{\text{7},i}$)
8. initiation age is less than 18 years ($\beta_{\text{8},i}$)
9. initiation age is 18 years or greater ($\beta_{\text{9},i}$)
10. White/Asian/Hispanic/other ($\beta_{\text{1}\text{0},i}$)
11. age in year *j* – 1 ($\beta_{\text{1}\text{1},i}$)
12. male ($\beta_{\text{1}\text{2},i}$)
13. living below poverty level ($\beta_{\text{1}\text{3},i}$)

For the ever-smoker model, smoking state 4 (non-menthol cigarette users) is used as the reference category.

The regression model equation is the same for never-smokers and ever-smokers, but there are differences in how it is used in the two cases. For never-smokers *i* will range over *i* = 1, 2, 3, 5. For ever-smokers *i* will only range over *i* = 2, 3, 5 because ever-smokers can never transition to never-smokers. In addition, all individuals in the never-smoker model have the same tobacco use history in the prior year and are currently never smokers so only the intercept term (θ) and demographic covariates are included in the model. Therefore, the equation for the multinomial regression model that estimates tobacco use transition probabilities for never-smokers is the following:


$$\;\;\;\;\;\;\;\;\;\;\text{ln}\left(\frac{p_{j,\;i}}{p_{j,\text{4}}}\right)=\;\gamma \;+\;\theta_{\text{1}}\times I\;\left(\text{Age}\;\text{when}\;\text{initiated}\;\text{smoking}\;<\text{1}\text{8}\right)\;$$

$$\;\;\;\;\;\;\;\;\;\;\;\;\;\;\;\;\;+\;\theta_{\text{2}}\times I\;\left(\text{Age}\;\text{when}\;\text{initiated}\;\text{smoking}\;\geq \text{1}\text{8}\right)$$

$$\;\;\;\;\;\;\;\;\;\;\;\;\;\;\;\;\;\;\;\;\;\;\;\;\;\;\;\;\;+\;\theta_{\text{3}}\times I\;\left(\text{R}\text{ace}\;=\text{White}/\text{Asian}/\text{Hispanic}/\text{Other}\right)$$

$$\;\;\;+\;\theta_{\text{4}}\times I\;\left(\text{Age}\;\text{in}\;\text{year}\;j\;-\text{1}\right)$$

$$+\;\theta_{\text{5}}\times I\;\left(\text{Sex}\;=\;\text{male}\right)$$

$$\;\;\;\;\;\;\;\;\;\;\;\;\;\;\;\;\;\;\;\;\;\;\;\;\;\;\;\;\;\;\;+\;\theta_{\text{6}}\times I\;\left(\text{Annual}\;\text{household}\;\text{income}\;\text{below}\;\text{poverty}\;\text{level}\right)$$ (A2)

The covariates and their associated parameters in the never-smoker model are the following:

1. initiation age is less than 18 years ($\theta_{\text{1}}$)
2. initiation age is 18 years or greater ($\theta_{\text{2}}$)
3. White/Asian/Hispanic/Other ($\theta_{\text{3}}$)
4. age ($\theta_{\text{4}}$)
5. male ($\theta_{\text{5}}$)
6. living below poverty level ($\theta_{\text{6}}$)

For ever-smokers and never-smokers, we denoted the logits as:


$$L_{j,i}=\text{ln}(\frac{p_{j,i}}{p_{j,\text{4}}})$$ (A3)

We obtained the transition probabilities from the logits using the following two equations:


$$p_{j,\text{4}}=\;\frac{\text{1}}{\text{1}+\;\sum_{i}e^{L_{j,i}}}$$ (A4)

$$p_{j,i}=\;p_{\text{4}}e^{L_{j,i}}$$ (A5)

#### Simulating mortality.

Mortality risk was simulated for each individual in each year of the simulation model. For individuals less than 55 years old, mortality risk in a given year was determined based on an individual’s age and sex. Mortality rates from NVSR Life Tables in 2016 [25] and 2017 [26] were applied in the simulation model in 2016 and 2017, respectively. Mortality rates from NVSR Life Tables in 2018 [27] were applied in 2018 and for all subsequent years in the simulation model. The model simulates mortality in the absence of coronavirus-2019 (covid-19) because at the time of model development life tables covering the period of the covid-19 pandemic were not available.

Among individuals 55 years and older, mortality risk in a given year was determined based on an individual’s age, sex, and current tobacco use state. We used data from Thun et al. [23] on mortality risk by smoker status (S1 and S2 Tables in S1 Text) and NHIS estimates of the prevalence of never, former and current smokers (S3 and S4 Tables in S1 Text) to estimate death rates for never, former, and current smokers while maintaining the average death rate by age and sex in the NVSR Life Tables. Among former smokers, mortality risk ratios in a given year were dependent on the number of years an individual had quit smoking. We used distinct mortality risk ratios for former smokers that had quit for less than 2 years, 2–4 years, 5–9 years, 10–19 years, 20–29 years, 30–39 years, 40–49 years, or greater than 50 years.

Informed by prior research, we assumed no difference in mortality risk between menthol cigarette and non-menthol cigarette smokers [28,29]. In addition, we assigned e-cigarette/dual users the same mortality risk as cigarette smokers because the majority of e-cigarette users also used cigarettes. Research indicates that exclusive e-cigarette users have lower levels of toxicant exposure than exclusive cigarette users, so mortality risk may be over-estimated for exclusive e-cigarette users in the model [30,31]. On the other hand, among dual e-cigarette and cigarette users our review of data from the PATH study indicate that these individuals used a similar quantity of cigarettes as compared to cigarette-only users, potentially minimizing benefits of e-cigarette use on reduced mortality.

#### Model outcomes.

In each year of the simulation model, we track the number of individuals that fall into each of the five tobacco use states (never smokers, former smokers, menthol cigarette smokers, non-menthol cigarette smokers, e-cigarette/dual users) and the number of individuals that die. For each model outcome, we provide mean estimates and 95% uncertainty intervals (ranges in which 95% of simulation results fell, across model replications).

#### Uncertainty, model calibration and validation.

To incorporate uncertainty into the simulation model, we drew 20 sets of parameter values for tobacco use-adjusted mortality relative risk from distributions characterized by Thun et al. [23] and created 20 versions of the initial population. We crossed each initial population with each tobacco use-adjusted mortality scenario, resulting in 400 total model iterations (20 X 20), each with distinct initial populations and mortality risk estimates. Each model iteration was replicated 25 times with different random number seeds, resulting in 10,000 total model replications.

**Fig 2:**
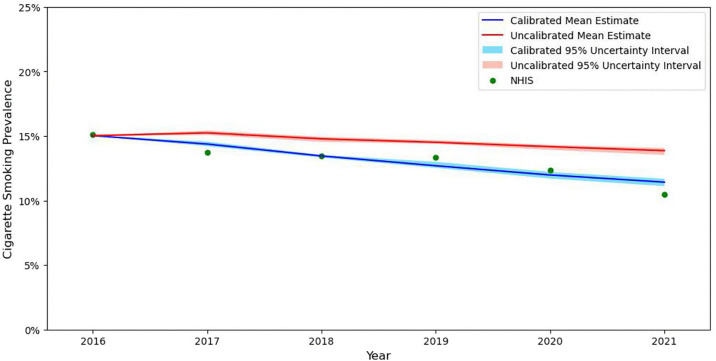
Prevalence of Cigarette Smoking Pre- and Post-Model Calibration, US Population 18-64 years. *Notes.* NHIS = National Health Interview Survey. Values presented from the simulation model are the estimate and corresponding 95% uncertainty interval.

We calibrated the simulation model because comparison of smoking prevalence estimates produced from the simulation model in 2017–2021 to smoking prevalence estimates from NHIS data over the same time period indicated that the PATH-informed simulation model over-estimated smoking prevalence (Fig 2). To calibrate the model to better match NHIS smoking prevalence estimates, we reduced initiation rates (i.e., percentage reduction in the probability that individuals who are never smokers transition to menthol, non-menthol, or e-cig/dual users) and increased cessation rates (i.e., percentage reduction in the probability of menthol, non-menthol, or e-cig/dual users transitioning to a former smoker in the subsequent year) each by 5.5% every year of the model. We calibrated each of the 10,000 model replications.

To validate the simulation model, we compared annual prevalence estimates of tobacco use from the calibrated simulation model to BRFSS (2016, 2017, 2021) and NHIS (2016–2021) prevalence estimates in the total adult US population and separately among individuals who are non-Hispanic Black and among those living above and below the poverty line. For model validation, we combined menthol and non-menthol cigarette smokers into a single category because the BRFSS and NHIS do not include questions about menthol cigarette use. We also compared prevalence rates of menthol cigarette use among individuals who smoke from the calibrated simulation model in 2016–2020 to prevalence estimates from NSDUH, which does include a survey item about menthol cigarette use over the same time period.

## Results

### Model validation

**Fig 3:**
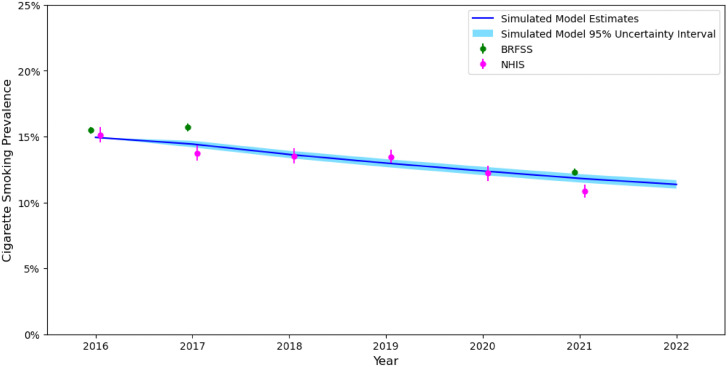
Cigarette Smoking Prevalence Estimates from the Simulation Model, BRFSS, and NHIS, US Population 18-64 years. *Notes.* Behavioral Risk Factor Surveillance System = BRFSS; National Health Interview Survey = NHIS. Values presented from the simulation model are the estimate and corresponding 95% uncertainty interval. Values presented from NHIS and BRFSS are the estimate and corresponding 95% confidence interval.

**Fig 4:**
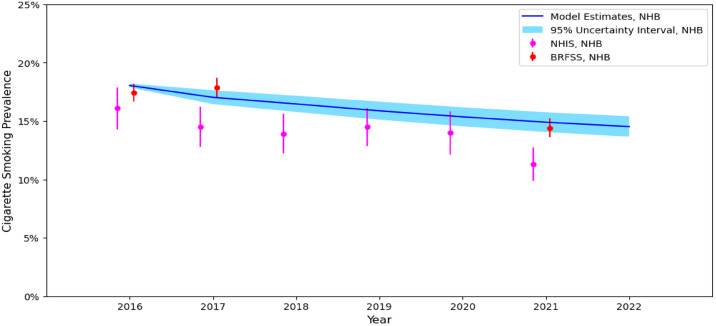
Cigarette Smoking Prevalence Estimates from the Simulated Model, BRFSS, and NHIS, non-Hispanic Black 18-64 years. Notes. NHB = Non-Hispanic Black; BRFSS = Behavioral Risk Factor Surveillance System; NHIS = National Health Interview Survey. Values presented from the simulation model are the estimate and corresponding 95% uncertainty interval. Values presented from NHIS and BRFSS are the estimate and corresponding 95% confidence interval.

The TwIST Model projects tobacco use over time among adults (18–64 years) living in the US. To validate the model, we compared modeled estimates of cigarette smoking prevalence in 2016–2021 to those from the nationally representative BRFSS and NHIS. In the US adult population, model projections of adult cigarette smoking (menthol + non-menthol) prevalence typically fell within the 95% confidence intervals of prevalence estimates from the BRFSS or NHIS (Fig 3). Among non-Hispanic Black individuals, model estimates typically fell within the 95% confidence intervals for estimates from the BRFSS, but modeled estimates were higher than NHIS estimates (Fig 4). Model estimates of e-cigarette/dual use were less consistent with estimates from the BRFSS and NHIS (S5 Table in S1 Text). Starting in 2018 our model underestimates the prevalence of e-cigarette/dual use as rates of e-cigarette use increased dramatically around that time. [32]

To assess estimates of menthol cigarette use, we compared modeled prevalence estimates of menthol cigarette use among cigarette smokers to those data from NSDUH (Table 2). In the adult population, model estimates fell within the 95% confidence intervals of NSDUH estimates in 2016 and 2017. Beginning in 2018, however, model estimates of menthol cigarette use were 3.9 to 6.1 percentage points higher than NSDUH estimates in the overall population. The model performed better among individuals who are Black or living in poverty. For these population groups, model estimates typically fell within 95% confidence intervals of NSDUH estimates or confidence/model uncertainty intervals overlapped.

**Table 2:** Prevalence Estimates of Menthol Cigarette Use among Cigarette Smokers.

	2016	2017	2018	2019	2020
*US Population*					
NSDUH	39.2 (37.8, 40.6)	40.1 (38.7, 41.5)	41.0 (39.5, 42.3)	42.0 (40.5, 43.5)	43.5 (40.6, 46.5)
Model	38.0 (37.8, 38.2)	41.4 (40.5, 42.4)	45.0 (43.8, 46.2)	47.5 (46.1, 48.8)	49.6 (48.1, 51.0)
*Non-Hispanic Black*					
NSDUH	87.4 (84.8, 89.9)	82.8 (79.7, 85.8)	86.4 (83.7, 89.1)	86.0 (83.0, 88.9)	80.4 (73.7, 87.1)
Model	82.8 (82.4, 83.2)	82.8 (80.6, 84.9)	84.8 (82.3, 87.2)	85.6 (82.8, 88.0)	86.1 (83.2, 88.7)
*Living in poverty*					
NSDUH	45.4 (42.7, 48.1)	47.0 (44.1, 50.0)	47.5 (44.5, 50.5)	52.6 (49.5, 55.7)	52.8 (47.1, 58.5)
Model	45.0 (44.7, 45.3)	48.4 (47.0, 49.9)	51.8 (50.1, 53.5)	54.2 (52.3, 56.1)	56.2 (54.2, 58.3)

NSDUH = National Survey on Drug Use or Health. Values presented from the simulation model are the estimate and corresponding 95% uncertainty interval. Values presented from NSDUH are the estimate and corresponding 95% confidence interval.

### Tobacco use projections

**Fig 5:**
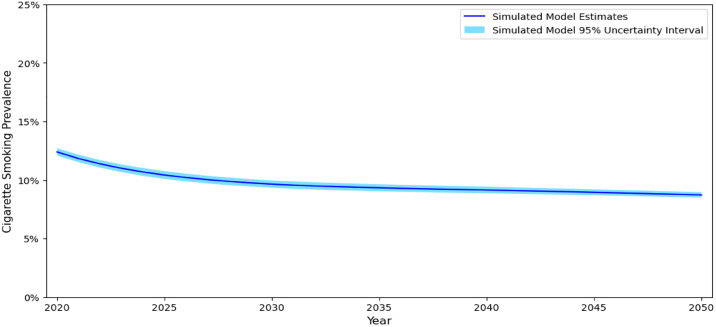
Simulated Cigarette Smoking Prevalence Estimates, US Population 18-64 years. Notes. Values presented are the estimate and corresponding 95% uncertainty interval.

**Fig 6:**
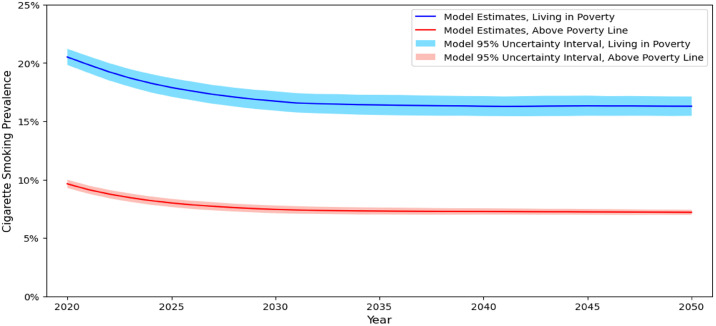
Prevalence of Cigarette Smoking by Poverty Status, US Population 18-64 years. *Notes.* Values presented are the estimate and corresponding 95% uncertainty interval.

Assuming the same tobacco control policy context over time, the simulation model estimates adult (18–64 years) cigarette (menthol and non-menthol cigarette users) smoking rates will decline from a prevalence rate of 12.4% (95% uncertainty interval [95% UI]: 12.1–12.7%) in 2020 to 9.6% ([95% UI]: 9.3–10.0%), 9.1% ([95% UI]: 8.9–9.4%), and 8.7% ([95% UI]: 8.5–9.0%) in 2030, 2040, and 2050, respectively (Table 3; Fig 5). The model also projects sustained income-based disparities in cigarette smoking (Fig 6). In 2020 individuals living in poverty had cigarette smoking rates 2.1 times as high as individuals living above the poverty line. From 2030 through 2050, adults living in poverty are estimated to have a cigarette smoking rate 2.2 times as high as individuals living above the poverty line.

**Table 3:** Modeled Prevalence Estimates of Tobacco Use, 2020 - 2050.

	2020	2030	2040	2050
*US Population*				
Cigarette Smoker	12.4 (12.1, 12.7)	9.6 (9.3, 10.0)	9.1 (8.9, 9.4)	8.7 (8.5, 9.0)
Menthol	6.1 (5.9, 6.4)	5.3 (5.1, 5.6)	5.0 (4.8, 5.2)	4.8 (4.6, 5.0)
Non-menthol	6.3 (6.0, 6.5)	4.3 (4.1, 4.6)	4.1 (3.9, 4.3)	3.9 (3.8, 4.1)
E-cig/Dual	2.1 (2.0, 2.2)	1.7 (1.6, 1.8)	1.7 (1.6, 1.8)	1.6 (1.5, 1.7)
*Non-Hispanic Black*				
Cigarette Smoker	15.4 (14.5, 16.2)	12.9 (12.0, 13.8)	12.3 (11.5, 13.1)	11.4 (10.7, 12.1)
Menthol	13.2 (12.5, 14.0)	11.1 (10.2, 12.0)	10.4 (9.6, 11.2)	9.5 (8.9, 10.2)
Non-menthol	2.1 (1.7, 2.6)	1.9 (1.4, 2.3)	1.9 (1.5, 2.3)	1.9 (1.6, 2.2)
E-cig/Dual	1.1 (0.8, 1.4)	0.9 (0.7, 1.2)	1.0 (0.7, 1.2)	0.9 (0.7, 1.2)
*All other race/ethnicities*				
Cigarette Smoker	12.0 (11.6, 12.3)	9.2 (8.9, 9.5)	8.7 (8.4, 9.0)	8.3 (8.1, 8.6)
Menthol	5.2 (4.9, 5.4)	4.5 (4.3, 4.8)	4.3 (4.1, 4.5)	4.1 (3.9, 4.3)
Non-menthol	6.8 (6.5, 7.1)	4.7 (4.4, 4.9)	4.4 (4.2, 4.7)	4.2 (4.1, 4.4)
E-cig/Dual	2.2 (2.1, 2.4)	1.8 (1.7, 1.9)	1.7 (1.6, 1.9)	1.7 (1.6, 1.8)
*Living in Poverty*				
Cigarette Smoker	20.5 (19.8, 21.2)	16.7 (15.9, 17.6)	16.3 (15.5, 17.2)	16.3 (15.5, 17.1)
Menthol	11.5 (11.0, 12.1)	10.3 (9.7, 10.9)	9.7 (9.1, 10.4)	9.5 (8.8, 10.1)
Non-menthol	9.0 (8.4, 9.5)	6.4 (5.9, 7.1)	6.5 (6.0, 7.2)	6.8 (6.3, 7.4)
E-cig/Dual	2.1 (1.8, 2.4)	1.4 (1.1, 1.6)	1.3 (1.1, 1.6)	1.4 (1.1, 1.6)
*Living Above FPL*				
Cigarette Smoker	9.6 (9.3, 10.0)	7.5 (7.1, 7.8)	7.3 (7.0, 7.5)	7.2 (7.0, 7.4)
Menthol	4.3 (4.1, 4.5)	3.8 (3.6, 4.0)	3.8 (3.6, 4.0)	3.8 (3.7, 4.0)
Non-menthol	5.3 (5.0, 5.6)	3.7 (3.4, 3.9)	3.5 (3.3, 3.7)	3.4 (3.2, 3.5)
E-cig/Dual	2.1 (1.9, 2.3)	1.8 (1.6, 1.9)	1.7 (1.6, 1.8)	1.7 (1.6, 1.8)

E-cig/dual = e-cigarette only and dual e-cigarette and cigarette user. FPL = federal poverty level. Values presented are the estimate and corresponding 95% uncertainty interval.

We also simulated the prevalence of menthol cigarette use over time (Table 3). From 2020 to 2050 the prevalence of non-menthol cigarette use is expected to decline at a higher rate than the prevalence of menthol cigarette use (21% vs. 38% decline). In addition, the model projects that non-Hispanic Black individuals and individuals living in poverty who smoke will continue to use menthol cigarettes at high rates. From 2020 to 2050 an estimated 83.7–86.0% of non-Hispanic Black individuals who smoke will use menthol cigarettes as compared to 43.0–49.4% among all other racial/ethnic groups. An estimated 56.2–61.5% of individuals living in poverty who smoke will use menthol cigarettes as compared to 44.8–53.3% of individuals living above the poverty line.

## Discussion

The TwIST Model is an individual-based model of tobacco use among the US adult population. The model can be used to estimate patterns of tobacco use over time and to help identify priority populations and potential opportunities for effective tobacco control intervention. In the setting of no meaningful change in tobacco policy, the model estimates that the prevalence of cigarette smoking will continue to decline, reaching rates of 9.6% and 8.7% among 18–64 year olds in 2030 and 2050, respectively. According to the model, cigarette smoking rates are not likely to meet the *Healthy People* goal of a 6% prevalence rate by 2030 [33]. The model also projects high rates of cigarette smoking among individuals living below the poverty line and that income-based disparities in smoking will persist. In addition, the majority of Black individuals and individuals living in poverty who smoke will continue to use menthol cigarettes. This is of concern because research indicates menthol cigarettes are more difficult to quit than non-menthol cigarettes, which may contribute to higher rates of tobacco-related disease in these groups [14,34].

The model suggests that existing tobacco control policies are not sufficient to reach ‘endgame’ smoking prevalence levels of 5% or less at the population level. To further progress, policies focused on regulating the tobacco retail environment such as caps on tobacco retailer density and bans on the sale of flavored tobacco products have been recommended as part of a comprehensive approach to tobacco control [35]. Tobacco retail policies such as these are also expected to reduce income-based disparities in tobacco use because tobacco retailer density, and consequently the availability and promotion of tobacco products, is disproportionately high in areas with a greater percentage of lower-income residents [35,36]. Such policies are already being implemented in some US cities and states [37,38]. For example, in 2015 San Francisco implemented a policy that caps the number of tobacco retailers in each of its eleven districts at 45 retailers [39]. Over time, the policy is expected to reduce tobacco retailer density the most in lower-income districts that are burdened by the highest number of tobacco retailers. [39] To date, at least 200 cities and two states (Massachusetts, California) ban the sale of menthol cigarettes [38]. Despite federal reports concluding that a menthol cigarette ban would improve the public health, in 2024 the White House decided to delay their decision on implementing a national menthol cigarette ban, and in 2025 the federal government withdrew the US Food and Drug Administration proposed rule to ban menthol cigarettes [40–42]. Research suggests that a menthol cigarette ban would promote smoking cessation, in particular among Black and lower-income adults who use the product the most [43].

Overall, comparisons of modeled estimates to those from national surveys indicate that the model projects cigarette smoking prevalence estimates that are similar to real-world estimates. The TwIST Model uses a 2^nd^ order Markov approach to simulate tobacco use over time. Prior research has found that higher-order Markov models that consider current *and* prior tobacco use better estimate future tobacco use behavior than 1^st^ order Markov models that only consider an individual’s current tobacco use [13]. Simulation models should consider incorporating higher-order Markov models to improve estimation of tobacco use behavior over time when data are sufficiently rich to allow this (i.e., sufficiently large cell sizes across modeled states).

Tobacco use trajectories in the simulation model were informed by transition probabilities between tobacco use states estimated from data in the PATH Study. The PATH Study is the largest nationally representative longitudinal study that tracks detailed information on tobacco use behavior in the US. PATH data are well suited for estimating trajectories of tobacco use in the total US population and in certain subpopulations with relatively large sample sizes in the data (e.g., individuals who use cigarettes and live below the poverty line). The PATH Study should consider oversampling priority populations for tobacco control with smaller sample sizes. To date, simulation models have focused on estimating the impacts of tobacco control policy interventions in the *total* US population. Large cohort studies that prioritize recruitment of priority populations for tobacco control are needed to inform model parameters to meaningfully estimate the impacts of public health interventions specific to each of them.

Although estimates of cigarette smoking from the simulation model typically matched those from national surveys, modeled estimates of e-cigarette/dual use were less consistent with BRFSS and NHIS estimates. The prevalence of e-cigarette/dual use may be underestimated in the simulation model because transition probabilities between tobacco use states were estimated using 2015–2017 data from the PATH Study. JUUL e-cigarettes were first introduced to the US market in 2015, and the prevalence of e-cigarette use continued to rise rapidly after 2017 [44]. As new tobacco products enter the market and prevalence rates of tobacco use change the model will need to be updated to reflect the evolving tobacco product landscape.

Overall, findings from this work suggest that the TwIST Model can be used to examine patterns of tobacco use in the adult US population. Projections from the model suggest that income-based disparities in cigarette smoking will persist. In addition, the prevalence of menthol cigarette use will increase over time under the current tobacco policy context. Policy change and programmatic efforts are needed to reduce income-based disparities in smoking and the rising prevalence of menthol cigarette use.

## Supporting information

S1 Text Supplement for TwIST model. (DOCX)
