# Supplementary material for: Development and validation of the Tobacco Use Individual-level Simulation and Tracking (TwIST) Model
Source: PLoS One. 2026 Feb 12;21(2):e0342083. doi: 10.1371/journal.pone.0342083 (PMC12900341; doi:10.1371/journal.pone.0342083)
Supplement: S1 Text — (DOCX) [file pone.0342083.s001.docx]

**Supplement for TwIST Model**

**Supplementary Table 1.** Relative risk of All-cause Mortality for Current Smokers versus Never Smokers

| **Age**  **(years)** | **Relative Risk Ratio, Men** | **Relative Risk Ratio, Women** |
| --- | --- | --- |
| 55-59 | 3.7 | 2.7 |
| 60-64 | 3.4 | 3.0 |
| 65-69 | 3.5 | 3.1 |
| 70-74 | 3.3 | 3.1 |
| 75-79 | 2.6 | 2.7 |
| 80-84 | 2.4 | 2.3 |
| 85+ | 1.8 | 1.9 |

*Source.* Thun et al.

**Supplementary Table 2.** Relative risk of all-cause mortality for former smokers versus current smokers

| **Years since quit** | **Relative Risk Ratio, Men** | **Relative Risk Ratio, Women** |
| --- | --- | --- |
| < 2 | 0.83 | 0.87 |
| 2-4 | 0.83 | 0.75 |
| 5-9 | 0.83 | 0.84 |
| 10-19 | 0.77 | 0.66 |
| 20-29 | 0.54 | 0.49 |
| 30-39 | 0.47 | 0.41 |
| 40-49 | 0.42 | 0.38 |
| 50+ | 0.39 | 0.37 |

*Source.* Thun et al. Among men, we applied the all-cause mortality risk ratio for individuals who have quit for 5-9 (RR = 0.83) years to individuals who have quit for less than less than 2 years and 2-4 years. We changed the risk ratios for those who have quit for less than 5 years so the all-cause mortality risk of former smokers is always lower than that of current smokers.

**Supplementary Table 3.** Smoking prevalence estimates of male never, former, and current smokers, 2018 National Health Interview Survey

| **Age** | **Never Smoker** | **Current Smoker** | **Former Smoker** |
| --- | --- | --- | --- |
| 55 | 57.67 | 18.06 | 24.26 |
| 56 | 57.22 | 18.45 | 24.33 |
| 57 | 52.51 | 20.69 | 26.80 |
| 58 | 53.84 | 22.61 | 23.56 |
| 59 | 50.52 | 18.27 | 31.21 |
| 60 | 55.71 | 15.75 | 28.54 |
| 61 | 46.77 | 22.16 | 31.07 |
| 62 | 51.16 | 16.16 | 32.68 |
| 63 | 51.72 | 15.69 | 32.59 |
| 64 | 45.91 | 12.98 | 41.11 |
| 65 | 53.78 | 11.67 | 34.55 |
| 66 | 47.00 | 16.99 | 36.01 |
| 67 | 41.64 | 15.02 | 43.33 |
| 68 | 43.48 | 12.89 | 43.63 |
| 69 | 37.32 | 12.15 | 50.53 |
| 70 | 38.75 | 11.43 | 49.81 |
| 71 | 42.25 | 11.46 | 46.28 |
| 72 | 38.19 | 10.23 | 51.58 |
| 73 | 36.26 | 8.64 | 55.11 |
| 74 | 31.13 | 12.61 | 56.26 |
| 75 | 44.90 | 7.78 | 47.32 |
| 76 | 42.79 | 1.28 | 55.93 |
| 77 | 39.16 | 4.39 | 56.45 |
| 78 | 29.78 | 3.84 | 66.37 |
| 79 | 42.84 | 8.15 | 49.01 |
| 80 | 32.86 | 8.10 | 59.04 |
| 81 | 33.55 | 7.91 | 58.54 |
| 82 | 42.10 | 1.85 | 56.05 |
| 83 | 28.01 | 8.05 | 63.94 |
| 84 | 27.36 | 4.28 | 68.35 |
| 85 | 43.30 | 4.88 | 51.82 |

**Supplementary Table 4.** Smoking prevalence estimates of female never, former, and current smokers, 2018 National Health Interview Survey

| **Age** | **Never Smoker** | **Current Smoker** | **Former Smoker** |
| --- | --- | --- | --- |
| 55 | 63.39 | 16.86 | 19.75 |
| 56 | 60.48 | 15.97 | 23.55 |
| 57 | 63.99 | 15.21 | 20.79 |
| 58 | 52.43 | 15.84 | 31.74 |
| 59 | 57.83 | 16.01 | 26.16 |
| 60 | 58.31 | 17.22 | 24.47 |
| 61 | 59.17 | 13.47 | 27.36 |
| 62 | 56.48 | 13.36 | 30.16 |
| 63 | 60.68 | 11.81 | 27.51 |
| 64 | 60.84 | 8.38 | 30.78 |
| 65 | 67.70 | 10.94 | 21.36 |
| 66 | 66.25 | 8.60 | 25.15 |
| 67 | 59.25 | 10.20 | 30.55 |
| 68 | 61.84 | 6.71 | 31.44 |
| 69 | 60.98 | 13.87 | 25.15 |
| 70 | 66.57 | 5.83 | 27.60 |
| 71 | 53.74 | 9.82 | 36.44 |
| 72 | 62.09 | 10.58 | 27.34 |
| 73 | 51.09 | 9.45 | 39.46 |
| 74 | 59.50 | 9.01 | 31.49 |
| 75 | 54.46 | 8.49 | 37.05 |
| 76 | 57.34 | 6.17 | 36.50 |
| 77 | 63.08 | 10.70 | 26.22 |
| 78 | 56.58 | 5.46 | 37.96 |
| 79 | 62.20 | 2.88 | 34.92 |
| 80 | 63.06 | 2.34 | 34.60 |
| 81 | 65.27 | 2.87 | 31.86 |
| 82 | 52.87 | 3.07 | 44.05 |
| 83 | 72.43 | 1.55 | 26.02 |
| 84 | 71.61 | 4.52 | 23.87 |
| 85 | 69.04 | 1.83 | 29.13 |

**Supplementary Table 5.** Prevalence of Tobacco Use from BRFSS, NHIS, and Simulation Model

|  | **2016** | | | **2017** | | | **2018** | | | **2019** | | | **2020** | | | **2021** | | |
| --- | --- | --- | --- | --- | --- | --- | --- | --- | --- | --- | --- | --- | --- | --- | --- | --- | --- | --- |
|  | BRFSS | NHIS | Model | BRFSS | NHIS | Model | BRFSS | NHIS | Model | BRFSS | NHIS | Model | BRFSS | NHIS | Model | BRFSS | NHIS | Model |
| *US Population* | | | | | | | | | | | | | | | | | | |
| Cigarette Smoker | 15.5 (15.3, 15.7) | 15.1 (14.5, 15.7) | 14.9 (14.9, 14.9) | 15.6 (15.4, 16.0) | 13.7 (13.2, 14.3) | 14.4 (14.2, 14.7) | - | 13.5 (12.9, 14.1) | 13.6 (13.4, 13.9) | - | 13.5 (12.9, 14.0) | 13.0 (12.7, 13.3) | - | 12.2 (11.7, 12.8) | 12.4 (12.1, 12.7) | 12.3 (12.1, 12.6) | 10.9 (10.4, 11.3) | 11.8 (11.5, 12.2) |
| E-cig/Dual | 5.4 (5.2, 5.6) | 3.7 (3.4, 4.0) | 3.7 (3.7, 3.7) | 5.2 (5.1, 5.5) | 3.3 (3.0, 3.7) | 3.0 (2.8, 3.1) | - | 3.9 (3.5, 4.2) | 2.6 (2.4, 2.7) | - | 5.4 (5.1, 5.8) | 2.3 (2.1, 2.4) | - | 4.6 (4.2, 5.0) | 2.1 (2.0, 2.2) | 8.1 (7.9, 8.3) | 5.6 (5.2, 6.0) | 1.9 (1.8, 2.1) |
| *Non-Hispanic Black* | | | | | | | | | | | | | | | | | | |
| Cigarette Smoker | 17.4 (16.6, 18.2) | 16.1 (14.3, 17.9) | 18.0 (17.9, 18.2) | 17.8 (17.0, 18.7) | 14.5 (12.8, 16.2) | 17.0 (16.4, 17.6) | - | 13.9 (12.2, 15.6) | 16.5 (15.8, 17.2) | - | 14.5 (12.9, 16.1) | 15.9 (15.1, 16.7) | - | 14.0 (12.1, 15.8) | 15.4 (14.5, 16.2) | 14.4 (13.6, 15.2) | 11.3 (9.9, 12.8) | 14.9 (14.1, 15.8) |
| E-cig/Dual | 3.9 (3.5, 4.4) | 2.2 (1.3, 3.1) | 1.9 (1.8, 2.0) | 3.6 (3.1, 4.1) | 2.3 (1.5, 3.2) | 1.6 (1.3, 1.9 | - | 2.1 (1.4, 2.8) | 1.4 (1.1, 1.7) | - | 4.0 (3.0, 5.0) | 1.2 (0.9, 1.5) | - | 1.8 (1.0, 2.5) | 1.1 (0.8, 1.4) | 6.1 (5.5, 6.6) | 2.7 (2.0 (3.5) | 1.0 (0.7, 1.3) |
| *All other race/ethnicities* | | | | | | | | | | | | | | | | | | |
| Cigarette Smoker | 15.2 (15.9, 16.4) | 15.0 (14.4, 15.6) | 14.5 (14.5, 14.5) | 16.2 (15.8, 16.5) | 13.6 (13.0, 14.2) | 14.1 (13.8, 14.4) | - | 13.5 (12.8, 14.1) | 13.3 (13.0, 13.6) | - | 13.3 (12.8, 13.9) | 12.6 (12.3, 12.9) | - | 12.0 (11.4, 12.6) | 12.0 (11.6, 12.3) | 12.8 (12.5, 13.0) | 10.8 (10.3, 11.3) | 11.4 (11.1, 11.8) |
| E-cig/Dual | 6.2 (6.0, 6.4) | 3.9 (3.6, 4.3) | 4.0 (3.9, 4.0) | 6.1 (5.9, 6.3) | 3.5 (3.1, 3.8) | 3.2 (3.0, 3.3) | - | 4.1 (3.8, 4.5) | 2.7 (2.6, 2.9) | - | 5.6 (5.2, 6.0) | 2.4 (2.3, 2.6) | - | 5.0 (4.5, 5.4) | 2.2 (2.1, 2.4) | 8.9 (8.7, 9.2) | 6.0 (5.6, 6.4) | 2.1 (1.9, 2.2) |
| *Living in Poverty* | | | | | | | | | | | | | | | | | | |
| Cigarette Smoker | - | 24.1 (22.3, 25.9) | 23.3 (23.1, 23.4) | - | 21.8 (19.9, 23.7) | 22.6 (22.1, 23.2) | - | 22.2 (20.2, 24.2) | 22.0 (21.3, 22.5) | - | 21.9 (20.0, 23.8) | 21.2 (20.5, 21.8 | - | 21.9 (19.6, 24.2) | 20.5 (19.8, 21.2) | - | 19.6 (17.6, 21.6) | 19.9 (19.2, 20.6) |
| E-cig/Dual | - | 3.6 (2.8, 4.5) | 4.7 (4.7, 4.8) | - | 4.2 (3.4, 5.0) | 3.5 (3.2, 3.8) | - | 4.6 (3.6, 5.6) | 2.9 (2.6, 3.2) | - | 6.2 (5.1, 7.4) | 2.4 (2.1, 2.7) | - | 3.8 (2.8, 4.8) | 2.1 (1.8, 2.4 | - | 7.1 (5.9, 8.4) | 1.9 (1.6, 2.2) |
| *Living Above FPL* | | | | | | | | | | | | | | | | | | |
| Cigarette Smoker | - | 13.7 (13.1, 14.3) | 12.0 (12.0, 12.1) | - | 12.5 (11.9, 13.1) | 11.6 (11.3, 11.9) | - | 12.3 (11.7, 12.9) | 10.8 (10.5, 11.1) | - | 12.4 (11.8, 12.9) | 10.2 (9.9, 10.5) | - | 11.1 (10.5, 11.7) | 9.6 (9.3, 10.0) | - | 9.8 (9.3, 10.3) | 9.1 (8.8, 9.5) |
| E-cig/Dual | - | 3.7 (3.4, 4.1) | 3.4 (3.4, 3.4) | - | 3.2 (2.9, 3.6) | 2.8 (2.6, 3.0) | - | 3.8 (3.4, 4.2) | 2.5 (2.3, 2.6) | - | 5.3 (4.9, 5.7) | 2.2 (2.1, 2.4) | - | 4.7 (4.2, 5.1) | 2.1 (1.9, 2.3) | - | 5.4 (5.0, 5.8) | 2.0 (1.8, 2.1) |

*Notes.* BRFSS = Behavioral Risk Factor Surveillance System; NHIS = National Health Interview Survey. FPL = federal poverty line. Values presented are the estimate and corresponding 95% uncertainty interval.
